# Social engagement revealed by gaze following in third-party observers of simulated social conflict

**DOI:** 10.3389/fpsyg.2022.952390

**Published:** 2022-12-12

**Authors:** Tess M. Champ, SeungHyun Lee, Anne B. Martin, Cameron M. Bolles, Sun Woo Kim, Katalin M. Gothard

**Affiliations:** ^1^Department of Physiology, College of Medicine, The University of Arizona, Tucson, AZ, United States; ^2^Department of Electrical and Computer Engineering, College of Engineering, The University of Arizona, Tucson, AZ, United States

**Keywords:** gaze following, joint attention, mentalizing, reflexive, saccade, macaque, social, naturalistic stimuli

## Abstract

Humans and non-human primates can allocate visual attention to areas of high interest in their visual field based on the behaviors of their social partners. Allocation of attention is particularly important for third-party observers of social interactions. By following the gaze of interacting individuals, the observer can obtain information about the mental states, emotions, and intentions of others. We presented three adult monkeys (*Macaca mulatta*) with videos of simulated social interactions and quantified their eye movements to determine which observed behaviors were most conducive to gaze following. Social interactions were simulated by juxtaposing two videos depicting a threatening and an appeasing individual facing each other, with the timing of the facial and bodily displays adjusted to mimic an exchange of social signals. Socially meaningful facial displays combined with full body movements significantly enhanced the probability of gaze following and joint attention. Despite the synthetic nature of these interactions, the facial and bodily displays of the submissive individual elicited significantly more joint-attention than gaze-following saccades, suggesting a preferential allocation of attention to the recipients of threatening displays. Temporal alignment of gaze following and joint attention to the frames of each video showed numerous clusters of significant increases in the frequency of these saccades. These clusters suggest that some videos contained signals that can induce a quasi-automatic redirection of the observer’s attention. However, these saccades occurred only on a fraction of the viewings, and we have documented large inter-individual variations. All viewers produced sequences of joint attention saccades (check-backs) shifting their attention between the two monkeys as though monitoring the simulated emitting-receiving cycle of social signals. These sequences reflect the viewer’s interest in monitoring the ongoing exchange of agonistic and affiliative displays. It appears that in macaque monkeys, the scanpaths of third-party observers of simulated social interactions are informed by social-cognitive processes suggestive of mentalizing.

## Introduction

Both humans and non-human primates exchange gaze-mediated social signals. They can redirect the visual attention of their social partners by inducing them to produce gaze-following and joint-attention saccades (e.g., Emery, 2000; Shepherd, 2010). *Saccades* are fast eye moments between two consecutive fixations. Between saccades, the eyes rest in *fixations* allowing the visual system to process the sensory details of the fixated target. The succession of fixations and saccades form a *scanpath* that contains information about the features of the visual scene that capture the viewer’s attention and sheds light on the viewer’s cognitive state. According to the operational definition proposed by Emery (2000), gaze-following saccades start from the eye or face of the observed individual and follow their gaze direction. Joint-attention saccades are a distinct subset of gaze-following saccades that land on the specific object that we infer is viewed by the observed individual (Emery, 2000; Shepherd, 2010). This definition does not require the saccades of joint attention to be preceded by eye contact or any other form of direct engagement between the observer and the observed individual. When gaze following and joint attention are preceded by eye contact or similar engagement, these eye movements are called shared attention (see Emery, 2000). Note that these definitions contain key elements but do not overlap in every detail with the canonical definitions emerging from work with human infants and with chimpanzees (Butterworth and Cochran, 1980; Tomasello, 1995; Povinelli and Eddy, 1996; Carpenter et al., 1998; Brooks and Meltzoff, 2005; Flom et al., 2007; Mundy and Newell, 2007; Carpenter and Liebal, 2011; Wellman, 2011; Leavens et al., 2019; Bard et al., 2021) however, these definitions are more appropriate to characterize the gaze behaviors of macaque third-party observers of simulated social interactions.

Gaze following and joint attention have been successfully used to infer social-cognitive skills in non-human primates (Tomasello, 1995; Emery et al., 1997; Tomasello et al., 1998; Deaner and Platt, 2003; Shepherd, 2010; Bettle and Rosati, 2019). Depending on the behavioral context, these gaze behaviors may represent simple orienting reflexes (Friesen and Kingstone, 1998; Emery, 2000; Penn and Povinelli, 2007) or social-cognitive processes such as mentalizing (reviewed by Arre and Santos, 2021). According to Frith and Frith (2006) “The term mentalizing was coined to refer to the process by which we make inferences about mental states. Much of the time these inferences are made automatically, without any thought or deliberation.” In the animal world, especially in species that live in complex, hierarchical societies, it is adaptive for individuals to infer the mental states of their social partners in order to anticipate their future actions. Indeed, gaze following and joint attention represent prerequisites for the ability to internally model the visual perspective of others and by extension, their knowledge, emotions, and intentions, known as ‘theory of mind’ (Baron-Cohen, 1995; Tomasello and Carpenter, 2007; Amici et al., 2009; Drayton and Santos, 2016).

There are at least two distinct social situations that elicit gaze following in macaques. In the canonical condition, the gaze follower is directly engaged by a social partner who is looking in the direction of an object or event of interest. For example, free ranging rhesus monkeys on the island of Cayo Santiago followed the gaze of a familiar human demonstrator who looked upward (Bettle and Rosati, 2019). In this case, the demonstrator actively recruited the attention of the gaze follower, who was engaged in a social interaction with the demonstrator. Compare this to the case where the gaze follower is a third-party observer of an exchange of social signals between two or more individuals. Here the gaze follower is not directly involved in the interaction, yet by monitoring the behaviors of others, the viewer gleans information about the observed individuals thereby vicariously gaining social experience. A classic example of third-party joint attention, without any of the observed individuals recruiting the viewer, was offered by Klin et al. (2002), who reported the scanpaths of viewers presented with a segment from the movie “Who’s afraid of Virginia Woolf?” In this segment, the wife of a man, who is visible in the background, was flirting with a younger man and insulting the husband. Unlike autistic viewers, neurotypical adults followed the gaze of the flirting pair, shown in the foreground, but also looked at the husband in the background, who was within hearing distance. The joint-attention saccades in the foreground marked allocation of social attention to the ongoing social exchange, while the saccades to the husband in the background indicated an empathetic response to his prerogative. Similar studies in macaques have shown that the scanpaths elicited by videos with ethologically valid social content contain information about social knowledge of the viewers (Mosher et al., 2011; Putnam et al., 2016).

The goal of our study was to assess the extent of social engagement in macaque third-party observers by characterizing their gaze-following and joint-attention saccades while they watched videos of simulated social conflict between conspecifics. We simulated dyadic conflict by juxtaposing two videos depicting a threatening and an appeasing individual oriented toward each other, with the timing of the facial and bodily displays adjusted to mimic an exchange of social signals. We chose to depict conflict because rhesus macaques live in despotic societies, where the strict social hierarchy is enforced through real or ritualized threats displayed by dominant individuals toward subordinates, who reciprocate the threats with submissive and affiliative signals (e.g., Vessey, 1984; Bernstein and Ehardt, 1985; Maestripieri and Hoffman, 2012). To the extent that awareness of the social status of others (often in relation to the social status of self) facilitates predicting aspects of the behaviors of others (Kliemann and Adolphs, 2018), status-dependent joint attention may qualify as a form of mentalizing in non-human primates.

A secondary goal was to distinguish between the reflexive and social cognitive mechanisms that underlie third-party gaze following and joint attention. In some circumstances gaze following is indeed merely reflexive (e.g., Emery, 2000), but recent studies have shown that gaze following in monkeys can reflect mentalizing and elements of theory of mind (Rosati et al., 2010; Krupenye and Call, 2019; Arre and Santos, 2021). For example, monkeys reflexively orient their gaze toward a target presented on the monitor in the line of sight of another monkey, who is shown in profile (Deaner and Platt, 2003). In contrast, mentalizing (theory of mind) is inferred when animals look where observed individuals, motivated by surprise, look at a novel item (Drayton and Santos, 2017). Check-back saccades, used by viewers to monitor the emitting-receiving cycle of social signals, and to explore the effects of these signals on the recipients, are also suggestive of mentalizing processes (Tomasello et al., 2001; Bräuer et al., 2005; Call and Tomasello, 2008). Our goal was to identify specific stimulus features that trigger either reflexive gaze behaviors or gaze behaviors indicative of the viewer’s ability to attribute mental states to others.

## Materials and methods

All experiments were performed in compliance with the guidelines of the National Institute of Health for the use of primates in research and were approved by Institutional Animal Care and Use Committee at the University of Arizona. All analyses were conducted using custom scripts in MATLAB R2021b (Mathworks) and OpenCV version 3.4 (Bradski, 2000).

### Subjects

We analyzed the gaze-following and joint-attention saccades of three adult rhesus monkeys: a male (D, 10 yo, 11.8 kg) and two females (C, 13 yo, 8.8 kg; P, 10 yo, 8.8 kg). All three monkeys were housed in double-sized cages, in the same room, on a regular light/dark cycle, with visual access to other monkeys in the colony. Monkeys C and P were cage mates. The animals benefitted from a daily enrichment schedule that included puzzles, toys, and foraging for fruits, nuts, and vegetables. The three monkeys were fitted with a three-pronged head fixation device attached to a cranial implant. The implantation was performed under Isoflurane anesthesia followed by post-operative pain management performed by the veterinary staff.

### Stimuli

**Figure 1:**
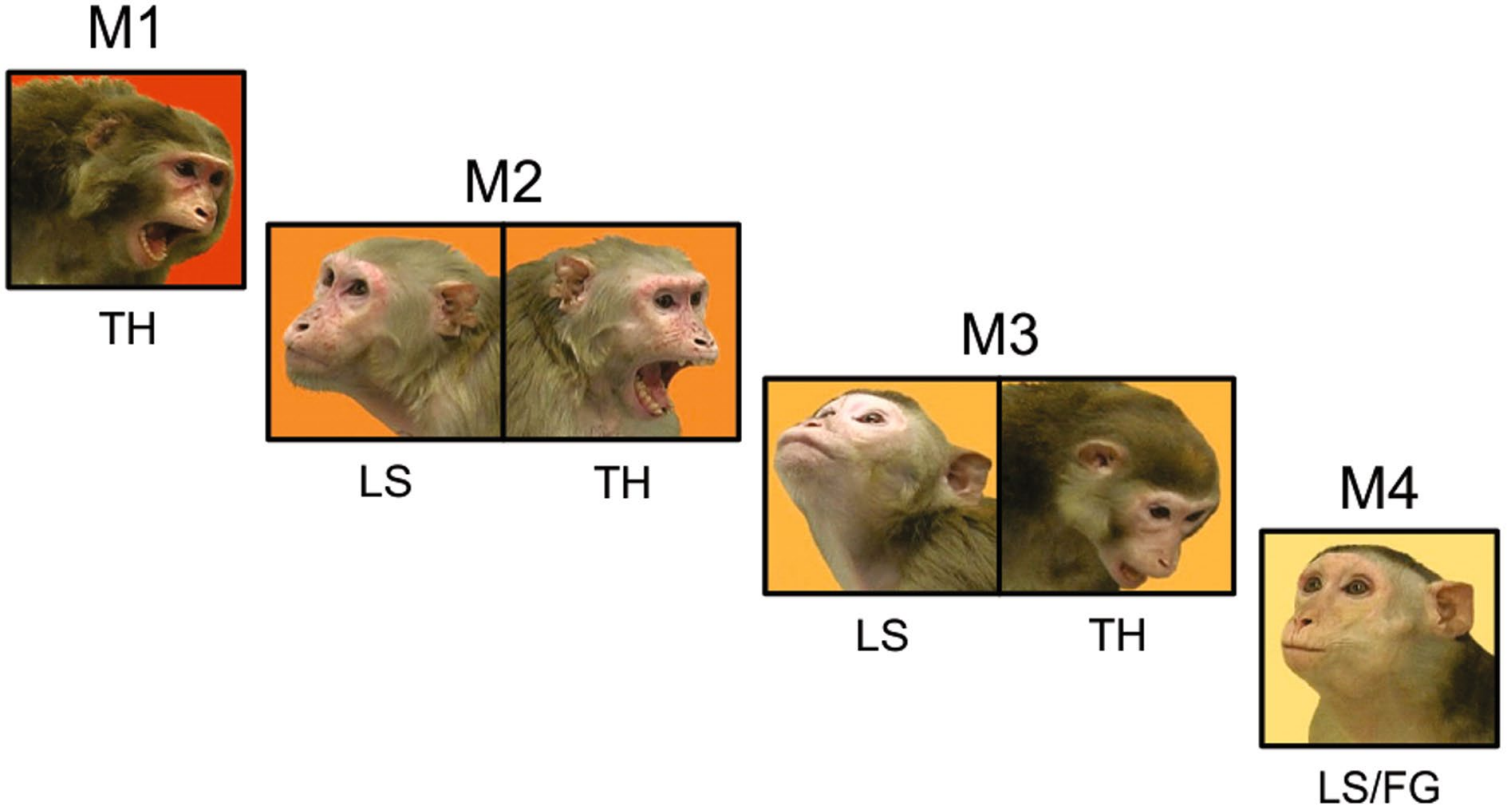
The inferred hierarchical structure of a video stimulus set. Each monkey in this set of four females is shown displaying behaviors that correspond to her position in the synthetic hierarchy. Three pair-wise interactions are shown (M1 with M2, M2 with M3, and M3 with M4). In the full video set, M1 was also paired with M3 or M4, and M2 was also paired with M4. Note that in all pairings, M1 was always shown threatening and M4 was always shown lipsmacking or fear grimacing. M2 was shown lipsmacking when paired with M1 and threatening when paired with M3 and M4. M3 was shown lipsmacking when paired with M1 and M2 and threatening when paired with M4.

We generated stimuli that we expected to induce gaze-following and joint-attention saccades using videos of natural macaque behaviors. We selected video segments that when juxtaposed would mimic agonistic social interactions between pairs of monkeys that were unfamiliar to the observer. In most videos, the body and face of the monkey had the same orientation; this redundancy allowed the preferential processing of social information from the body (Bliss-Moreau et al., 2017) to ameliorate some of the imperfections of the simulated social interactions. On one side of these juxtaposed videos an aggressive animal displayed an open-mouth threat in the direction of the animal on the other side, which displayed a lipsmack or fear grimace. The facial displays were timed to suggest an interaction where the subordinate appeared to respond with a submissive or affiliative facial expression to the threat received from the more dominant animal. While lipsmacking is not a purely submissive behavior, but rather an affiliative signal, we grouped lipsmacks and fear grimaces (silent bared teeth display) together to contrast with the threatening displays that are expected from dominant animals. To further enhance the ethological validity of these stimuli the animals shown in the videos were grouped in three synthetic hierarchy sets. Each set had 4 individuals of the same sex that allowed for 6 pairwise interactions. The animal at the top of the hierarchy, M1, was shown displaying an open mouth threat in all pairings, suggesting that it was the highest ranking in the group (Figure 1). The animal at the bottom of the hierarchy, M4, was shown displaying a lipsmack or a fear grimace (silent bared teeth) in all pairings, suggesting the lowest rank in the group. Monkeys in the middle, M2 and M3 were shown lipsmacking to the higher-ranking animals (M1 and M2 respectively) and threatening to the lower ranking animals (M3 and M4 respectively). This element of inferred hierarchy was intended to underline the agonistic interaction between the pairs of animals, however, we did not test the understanding of the inferred hierarchy by the observers.

Although the video segments were juxtaposed to create the impression of an interaction, they were imperfect in simulating mutual gaze. The two monkeys appeared oriented toward each other and the facial expressions were aligned in time, but a hard criterion of mutual gaze was not established. Even under natural conditions, social interactions do not require mutual gaze or face-to-face orientation (Kendon, 1967). Therefore, this aspect of the videos was not codified or quantified.

The resolution of the video stimuli was 1920 × 1080 full-HD; the scene on each side had a resolution of 640 × 480 VGA. Videos were displayed at 25 frames/s. Eye movements were recorded with an infrared eye tracker (ISCAN ETL-200) at a sampling rate of 120 Hz. A scanpath was generated from the X and Y coordinates of the eye movements, which were sampled at 1KHz and recoded using one of two data acquisition systems, an OmniPlex System (Plexon Inc.) or a Spike2 System from Cambridge Electronics Design (CED). Eye tracker calibration and video timing was controlled by one of two experimental control systems, Presentation (Neurobehavior Systems) or Monkey Logic.^1^ At the beginning of each session the eye position was calibrated. Each trial started with a fixation of 250 ms on the start cue, a 20 × 20 pixel white square with a 100 × 100 pixel error boundary in the center of the monitor, followed by the display of a video showing the face-to-face simulated conflict. The viewer monkey was allowed to freely scan the video or to look away. At the end of the video display (duration of 15 s, 375 frames) the subjects received juice reward. The videos were presented in blocks of 12 trials in which each of the 6 pairwise interactions were presented. The side of the video with the threatening monkey was counterbalanced. Each session consisted of at least 3 blocks. If the observer watched the videos less than 50% of the time in a session, that session was excluded.

### Identification of gaze-following and joint-attention saccades

**Figure 2:**
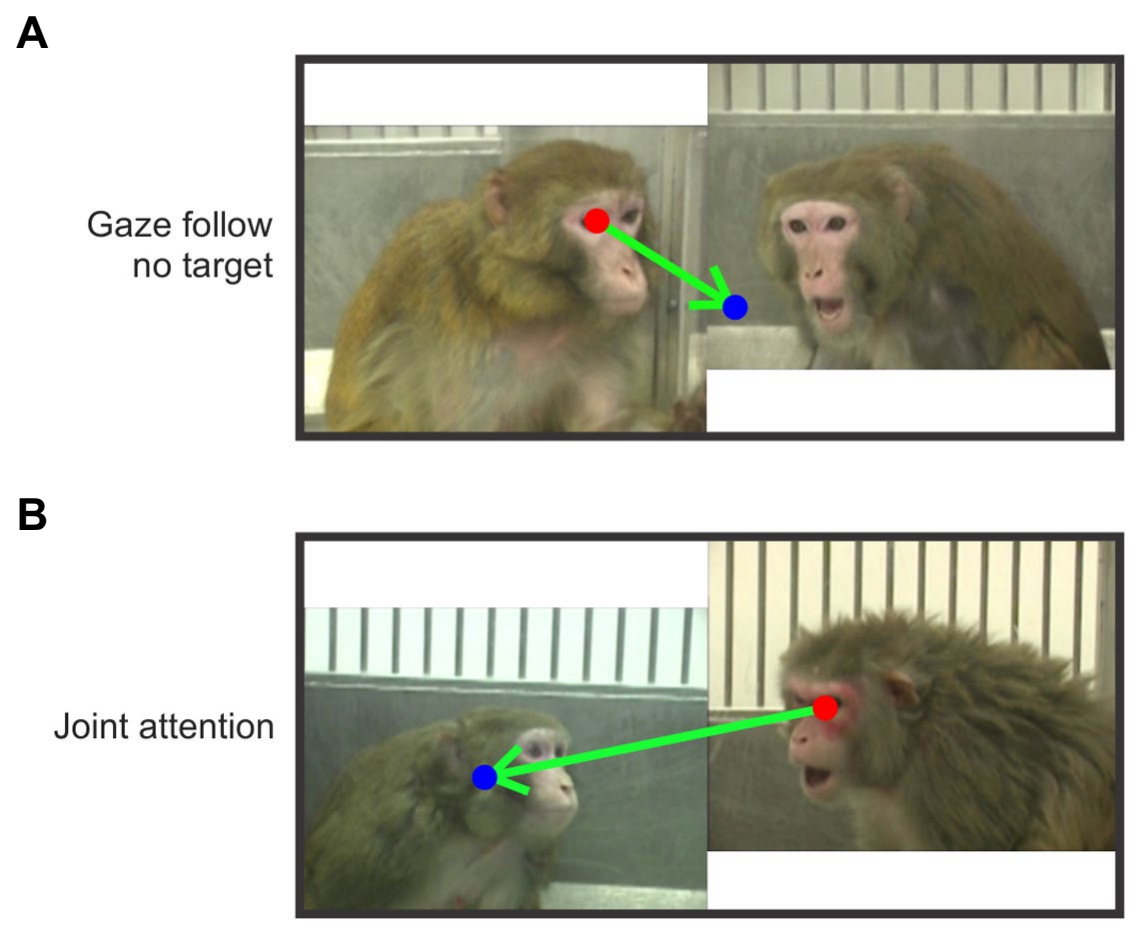
Examples of gaze-following and joint-attention saccades. The arrows indicate example saccades (start and end at the red and blue dot respectively). **(A)** An example of a gaze-following saccade that started from the eye of the subordinate monkey and did not land on the inferred target (the dominant monkey). **(B)** A joint-attention saccade that started from the eye of the dominant monkey and landed on the face of the subordinate monkey.

We identified gaze-following and joint-attention saccades in each trial by frame (hereafter referred to as JAGF saccades). We projected the mean eye position per frame to generate a smoothed scanpath. Using these smoothed scanpaths, JAGF saccades were manually scored and categorized based on the following criteria. *Gaze-Following saccade* (GF): a saccade that originated at the eye or face of one monkey, fell within ±30 degrees of the eye direction of the monkey, and landed outside the face or body of the other monkey. A gaze-following saccade is, therefore, a saccade without a target (Figure 2A). *Joint-Attention saccade* (JA): a saccade that originated at the eye or face of one monkey, fell within ±30 degrees of the eye direction of the monkey, and landed on the face or body of the other monkey (Figure 2B). These definitions are based on Emery (2000). Scanpaths were scored by two independent scorers and only saccades that were reconciled between them were included.

### Identification of frames that contained facial expressions and the exclusion of frames where gaze-following and joint-attention saccades were not possible

For each video monkey, we identified frames when the monkey made a facial expression (ethogram). The ethogram allows us to see when threatening and appeasing facial expressions co-occurred during the video; even if these facial expressions co-occurred, the videos were constructed such that the onset of the threat typically preceded the onset of the appeasing facial expression. We also classified frames when they were facing their partner, when they were facing the observer (3D), or when their face or eyes were not visible. The videos contained 26 sets of frames that were scored as 3D (699 frames total). The combinations of these factors allowed us to categorize frames wherein a JAGF saccade was not possible (e.g., the video monkey was facing the viewer, or the face wasn’t visible). To generate ethograms, at least three experimenters scored, frame-by-frame, each movie. Each movie had 375 frames and scoring reliability rates were expressed as Cohen’s Kappa: video set 1: 0.65; video set 2: 0.66; video set 3: 0.82. These values indicate that inter-scorer reliability is substantial for two video sets (>0.6) and almost perfect for one video set (>0.8) according to Landis and Koch (1977). We classified 10.4% of frames where a JAGF saccade was not possible.

We compared the rate (in Hz) of JAGF saccades initiated from a video monkey in frames when they made a facial expression (FE) and in frames without a facial expression (noFE):

$$JAGF_{FE}=\frac{\sum_{frame}\left(JAGF\&FE\right)}{\sum_{frame}FE}\times \frac{1\;frame}{40\;ms}$$

$$JAGF_{noFE}=\frac{\sum_{frame}\left(JAGF\&noFE\right)}{\sum_{frame}noFE}\times \frac{1\;frame}{40\;ms}$$

We also compared the proportion of joint-attention saccades over all JAGF saccades (JA%) when the saccade was initiated from dominant monkeys (D) compared to subordinate monkeys (S):

$$JA{\%}_{D}=100\times \frac{\sum_{frame}\left(JA_{D}\right)}{\sum_{frame}\left(JA_{D}+GF_{D}\right)}$$

$$JA{\%}_{S}=100\times \frac{\sum_{frame}\left(JA_{S}\right)}{\sum_{frame}\left(JA_{S}+GF_{S}\right)}$$

If one of these groups (all D compared to all S) had, by chance, more time facing away from their partner, the proportion of gaze-following saccades might be artificially inflated for that group. To account for this possibility, we computed the JA% using only frames when the video monkeys were facing each other, that is, we excluded 3D and looking away frames. We also computed the proportions including frames when they were facing away and found no difference in our conclusions. For both the FE vs. noFE and D vs. S comparisons, we used a chi-square test of proportions.

### Identification of windows with clustering of gaze-following and joint-attention saccades

To identify windows with significant clustering of JAGF saccades, i.e., times in the video that consistently resulted in gaze-following and joint-attention saccades, we used a non-parametric statistical analysis (based on the method of Maris and Oostenveld, 2007). We generated rasters of saccades by frame and trial for each of the 6 different interactions between the 4 members of a hierarchical set (e.g., M4 lipsmacking when paired with M1 threatening). Histograms of saccade counts per frame were generated for each observer and individual movie monkey, combined across partners. Thus, each histogram shows the clustering (or not) of gaze-following and joint-attention saccades generated by an observer monkey in response to one video monkey. We smoothed the z-scored histograms of these saccades (gaussian kernel with 2 frame sigma, z-score relative to mean and standard deviation across frames). Next, we generated a distribution of null histograms where the frame of each saccade was randomized in each trial, repeated 10,000 times. For each resulting histogram, we identified candidate clusters of saccades by comparing the smoothed histogram z-scores to the 95% confidence interval of the total null distribution. The null statistic for each histogram was defined as the maximum of the summed z-scores in each candidate cluster. The distribution of null cluster statistics was then compared to the cluster statistics from the veridical histograms, and clusters outside the 95% confidence interval of the null cluster distribution defined windows with significant clustering of saccades at *p* < 0.05.

For each observer, in each video we identified clusters of JAGF saccades initiated from each video monkey with all partners combined. For example, we identified clusters of JAGF saccades made by viewer C from M1 when paired with M2, M3, and M4. Note that the M1 video segments are identical for all three pairings. We also identified clusters of JAGF saccades from all partners to the same video monkey (e.g., clusters of JAGF saccades originating from M2, M3, and M4 and ending on M1).

We determined the proportion of frames that were part of clusters in all three observers, allowing us to quantify the degree of inter-observer variability in gaze behavior. We also determined the proportion of frames where two monkey’s clusters overlapped, and frames where only one monkey had clustering of JAGF saccades.

$$overlapproportion=\frac{numberofoverlappingclusterframes}{numberofclusterframes}$$

### Check-back saccades

We defined check-backs as sequences of 2 or more joint-attention saccades that were followed immediately (within 400 ms) by joint-attention saccades in the reverse direction, i.e., a rapid shift of attention from one video monkey to the other, and then back. We also determined the proportion of check back frames that were found within the clusters of JA saccades and whether the check-backs were originating more often from the subordinate or dominant individual.

### Frequency of gaze-following and joint-attention saccades within significant clusters

To determine how frequently JAGF saccades were produced by our observer monkeys, we used windows that contained clustering of such saccades. We computed the saccade frequency (in Hz):

$$frequency=\frac{saccadecountinclusters}{viewingtimeofclusterframes\;\left(seconds\right)}$$

### Identification of predictive or anticipatory gaze behavior

Given that the viewer monkeys watched each video multiple times, it was important to determine whether the monkeys learned the sequence of behaviors in the videos. Sequence learning would result in JAGF saccades occurring earlier in a cluster after repeated viewing of the same sequence of frames, eventually leading to anticipating the stimuli that elicit gaze following. Within a cluster, a single trial might have multiple JAGF saccades, so we marked the frame that contained the first JAGF saccade in each trial. We found the Pearson correlation coefficient of these frames over viewings in each cluster. For each viewer monkey we then used a t-test to determine whether the distribution of these rho values was shifted towards a negative mean, which would indicate that over multiple viewings the first JAGF saccade was likely to occur at earlier frames in each cluster. Mean rho values indistinguishable from zero were taken to indicate that this type of predictive behavior wasn’t present.

### Identification of movement within videos

**Figure 3:**
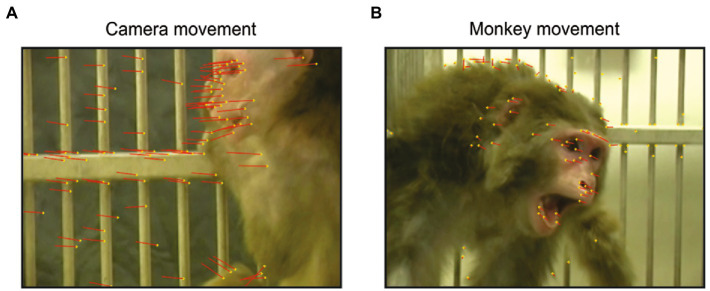
Examples of the two types of movement in a video frame. The feature points of each frame are shown in yellow dots, and the optical flow vectors are shown as red lines, indicating the translation of each feature point relative to the previous frame. **(A)** Optical flow produced by camera movement. **(B)** Optical flow produced by movement of the video monkey.

The videos contained two types of movement: movements made by the camera causing all objects in the background and foreground to shift (Figure 3A), and movements made by the video monkeys (Figure 3B). The optical flow analysis was a necessary control to eliminate the possibility that visual motion alone, other than body and face movements of social signaling, captured the attention of the viewer monkeys. Such visual motion could be generated by camera panning or zooming. Motion of either type was quantified by optical flow analysis (as in Lucas and Kanade, 1981). Identified feature points were used to measure optical flow and the translation of each point relative to the previous frame, yielding the optical flow vectors in that frame. We used the Shi-Tomasi corner detector algorithm to identify the feature points (Shi and Tomasi, 1994). The number of feature points in a frame (N), yield N optical flow vectors in the X direction ( $OF_{X}$ ) and the Y direction ( $OF_{Y}$ ). We defined the optical flow value, which is the amount of motion in a frame, as the mean of the magnitude of the optical flow vectors:

$$Optical\;Flow_{frame}=\frac{\sum_{n=1}^{N}\sqrt{{\left(OF_{Xn}\right)}^{2}+{\left(OF_{Yn}\right)}^{2}}}{N}$$

To remove noise from the optical flow results, we smoothed the signal using the Savitzky–Golay filter (Matlab function *smoothdata*), which increases the precision of the data without distorting the signal tendency. We set boundaries to capture the upper and lower 25%, which correspond to 0.67 standard deviations from the mean assuming the optical flow was normally distributed.

$$Movementamount=\{\begin{array}{c} High\;\left(when\;OF_{frame}\geq \mu_{OF}+0.67\sigma_{OF}\right)\;\\ Low\;\left(when\;OF_{frame}\leq \mu_{OF}-0.67\sigma_{OF}\right)\\ Moderate\;\left(Otherwise\right) \end{array}$$

## Results

From the three viewer monkeys we analyzed 3,743 trials (where a trial is a single viewing of a video, Figure 1) and identified 14,686 saccades that met our criteria for gaze following or joint attention, referred to here as JAGF saccades (Figure 2). Viewer monkey C watched three video sets over the course of 40 sessions for a total of 1,440 trials. Viewer monkey D also watched three video sets over the course of 38 sessions for a total of 1,368 trials. Viewer monkey P watched two sets over the course of 26 sessions, for a total of 935 trials. Individual video monkeys were watched on average 156 times (viewer D: 152, min = 66, max = 252; C: 160, min = 72, max = 270; P: 156, min = 60, max = 288). None of the three monkeys showed a significant decrease in looking time over the course of repeated viewing of the videos, perhaps because the viewings were separated by multiple days (8 video sets, paired t-test of first and second half of viewings, *p* = 0.3).

### Facial expressions, but not movement-related cues, elicit a higher rate of gaze-following and joint-attention saccades

The most reliable cues that elicit reflexive gaze-following saccades are shifts in eye, head, or body direction (Emery, 2000; Paukner et al., 2007; Tomasello et al., 2007; Shepherd, 2010). We computed the optical flow in each video frame and compared the occurrence of gaze-following and joint-attention saccades in frames with higher and lower optical flow. For viewer monkeys D and C, the likelihood of such saccades was lower when the optical flow was high (Observer D: 15.5% of gaze-following and joint-attention saccades occurred during high movement, 44% during low movement, chi-square *p* < 0.001, *n* = 152. Observer C: 14% of gaze-following and joint-attention saccades occurred during high movement, 43% during low movement, *p* < 0.001, *n* = 154). Viewer monkey P showed the opposite trend (44% of gaze-following and joint-attention saccades occurred during high movement, 11% during low movement, *p* < 0.001, *n* = 86). Thus, we found no consistent relationship between the amount of movement in the video and the likelihood of gaze-following and joint-attention saccades.

**Figure 4:**
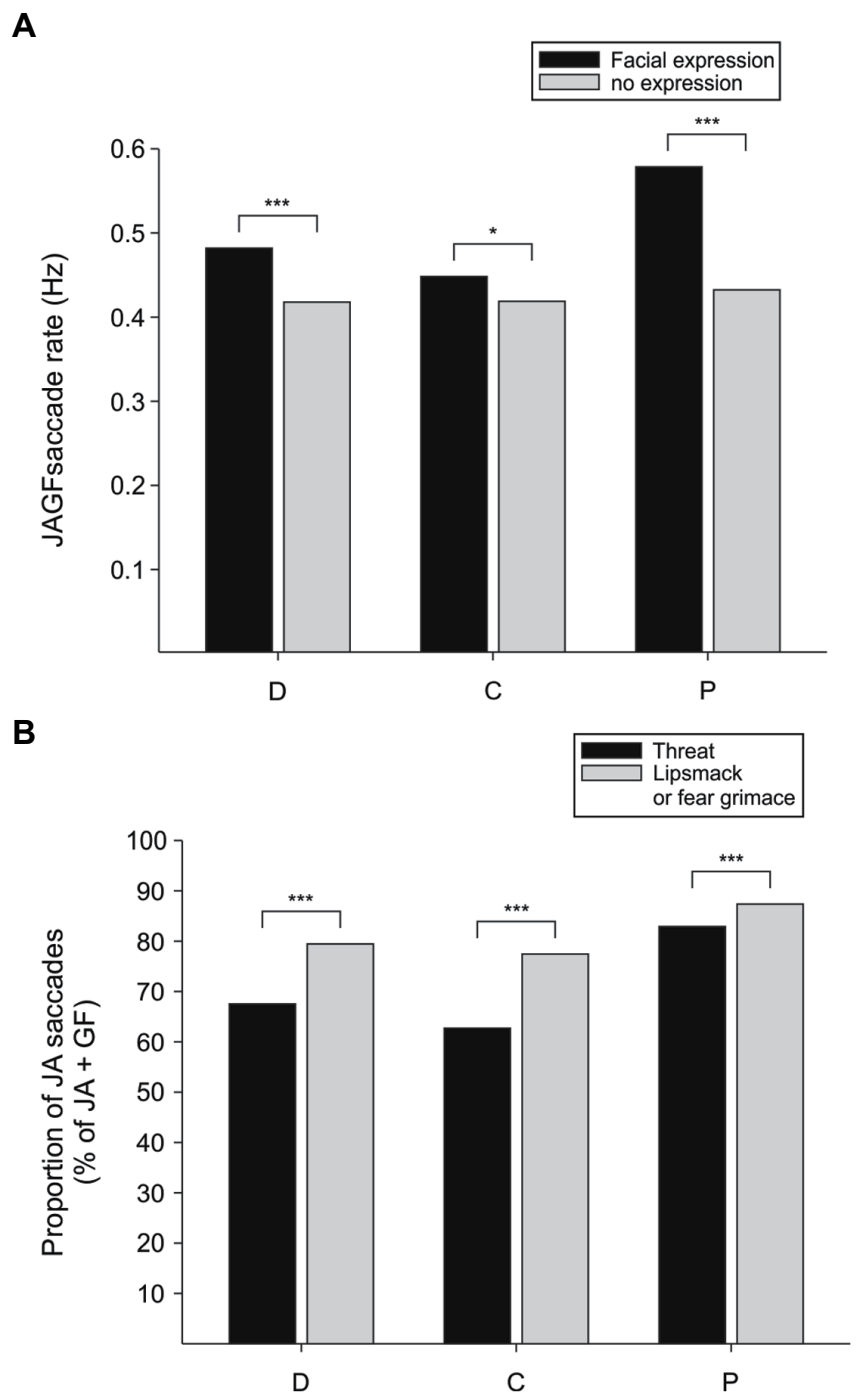
The probability of gaze following and joint attention is enhanced by facial expressions. **(A)** The rate of joint-attention and gaze-following (JAGF) saccades is higher in all three viewer monkeys (D, C, and P) during frames that show facial expressions. **(B)** Out of all JAGF saccades, a higher proportion of joint-attention (JA) saccades was elicited by the subordinate individuals in all three viewer monkeys.

Next, we compared the frequency of gaze-following and joint-attention saccades initiated from video monkeys in frames that contained facial expressions and frames in which the monkey had a neutral face. In all three observers, the rate of gaze-following and joint-attention saccades was higher in frames when the video monkey was making a facial expression (chi-square test of proportions, observer D: *p* < 0.0001, *n* = 5,154; C: *p* < 0.05, *n* = 4,772; P: *p* < 0.0001, *n* = 4,259; Figure 4A). Even though we do not directly measure what the observer knows, the increase in JAGF saccades when the observed video monkey makes a facial expression suggests that the observer at least attends to the emotional states of the observed individuals and is engaged in evaluating the target of their attention.

### Viewer monkeys preferentially follow the gaze of the subordinate monkeys

If the viewer monkeys simply divide their attention between the two sides of the video, looking back and forth between the two animals, there should be no systematic preference of joint-attention (JA) saccades originating from either video monkey. Instead, we found that whether the video monkeys were producing facial expressions or not, saccades that originated from the subordinate animals were more likely to land on the inferred target, the dominant monkeys (Figure 4B). In contrast, gaze following saccades that did not land on the target (GF) were more often originating from the dominant monkey. For all three observers, out of all JAGF saccades the proportion of joint-attention saccades was higher when starting from the subordinate monkey in the pair (JA/JAGF subordinate vs. dominant, chi-square test of proportions, viewer D: *p* < 0.0001, *n* = 4,747 frames; C: *p* < 0.0001, *n* = 4,499; P: *p* < 0.0001, *n* = 4,069). These findings suggest a preferential shift of social attention from the subordinate to the dominant animal. Joint-attention saccades reflect a higher cognitive process (deictic signal processing) that requires combining egocentric and allocentric spatial reference frames (Shepherd, 2010). A higher proportion of these saccades from the subordinate social partner indicates a mentalizing-like process that coordinates the viewer’s social attention. This outcome confirms that in macaques, facial expressions modulate gaze-following and joint-attention saccades not only when animals are directly engaged by demonstrators in their natural environment (Bethell et al., 2012) but also in the laboratory in response to simulated social interactions.

### The temporal distribution of gaze-following and joint-attention saccades show reliable maxima aligned to specific observed behaviors

**Figure 5:**
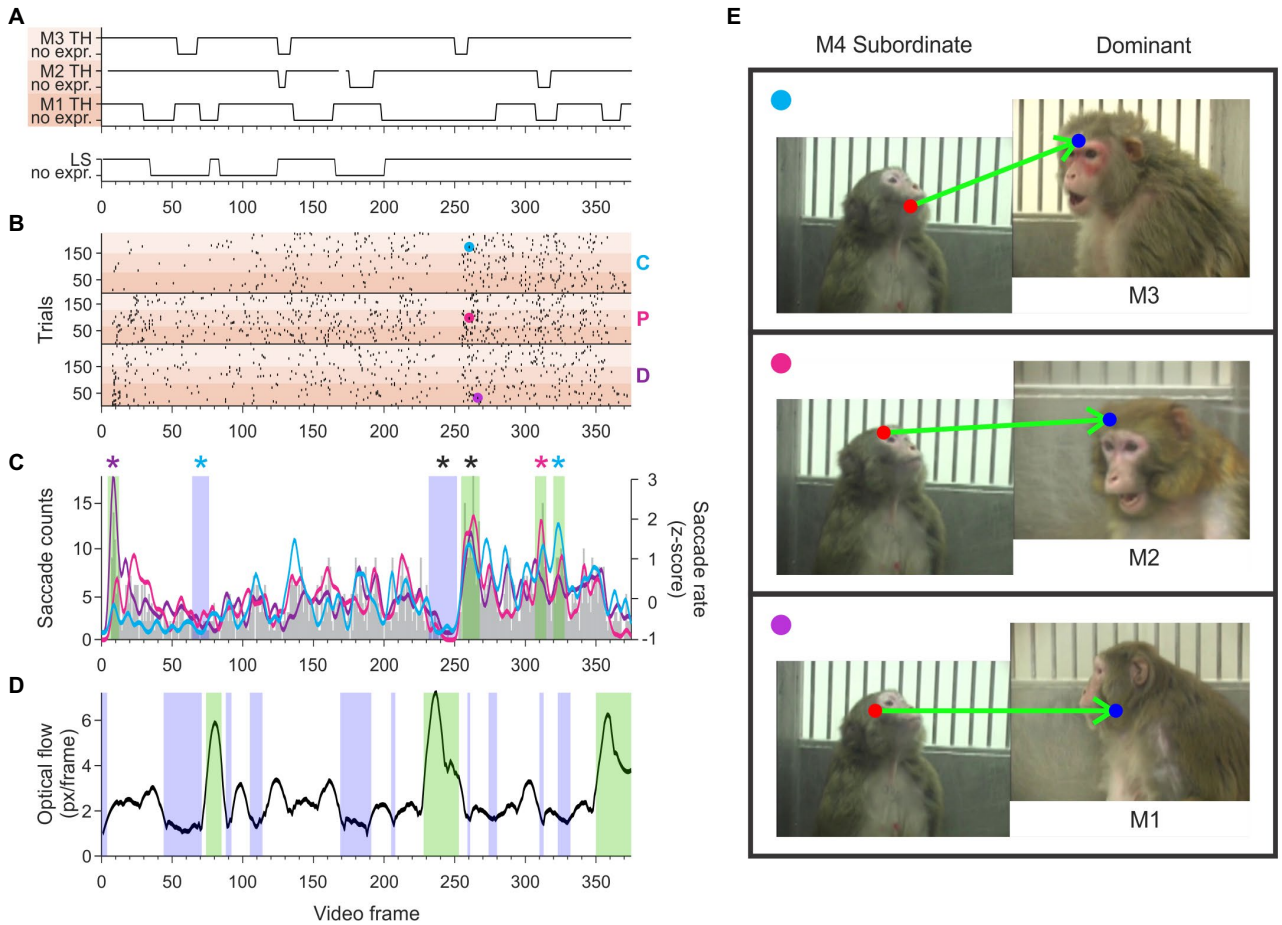
The temporal distribution of gaze-following and joint-attention saccades starting from a subordinate monkey M4. **(A)** The ethogram of the videos depicting the dominant partners M1, M2, and M3 (top) and the subordinate monkey M4 (bottom) indicates that the video contains frames depicting facial expression and neutral faces. Darker-to-lighter shading indicates partner monkeys M1-M3, all dominant, with the darker color indicating higher status. Gaps in the continuous line of the ethogram for Monkey 2 indicate frames when her face was not visible. **(B)** Combined rasters of JAGF saccades produced by viewers D, P, and C originating from the eyes and face of M4 interacting with M1, M2, and M3. Darker-to-lighter shading indicates monkey M1-M3. Cyan, magenta, and purple circles indicate the specific saccades shown in E. A gap in gaze-following and joint-attention saccades before frame 250 (when M4 was facing the viewer) was followed by a cluster of peak occurrences. **(C)** Histogram of saccade counts per frame across all three observers and all three partners (gray bars, left axis), and smoothed z-scored JAGF saccade rate of each observer (cyan: observer C; magenta: observer P; purple: observer D; right axis). Blue and green shading indicates windows with significant decrease or increase in JAGF saccades, respectively. Black stars indicate windows where all three viewers had significant clustering. Colored stars indicate the significant clustering by individual observers. **(D)** Optical flow in the video caused by the movement of the camera and/or the monkey. The blue and green shading indicate windows with significantly less and more movement than average, respectively. Note that video segments with increased movement do not overlap with clusters of gaze-following and joint-attention saccades. **(E)** Example saccades made by the three observers during the cluster of increased JAGF saccades around frame 250. The start and end of the saccades are indicated by a red and blue dot, respectively. Top panel: JA saccade of viewer monkey C, originating at subordinate monkey M4 landing on the brow region of dominant M3. Middle panel: JA saccade of viewer monkey P, originating from subordinate M4 landing on the forehead of M2. Bottom panel: JA saccade of viewer monkey D, originating from subordinate M4 and landing on the neutral face of M1.

We determined whether the timing of gaze-following and joint-attention saccades were equally distributed during the video or showed peaks and valleys aligned to specific behaviors. We identified clusters of frames (‘hot spots’) with a higher probability of eliciting JAGF saccades than what would be expected by a random distribution of these saccades (see Methods 2.5). Figure 5 shows an example of hot spots of gaze-following and joint-attention saccades originating from the eyes or face of the lowest ranking monkey (M4) in a female hierarchy. In each video, M4 is shown lipsmacking in the direction of three different threatening monkeys of presumed higher status (M1, M2, and M3). The ethogram in Figure 5A shows the timing of the threatening behavior from M1, M2, and M3 (top three traces marked by gradually darker shading) relative to the lipsmacking behavior (bottom trace) from M4. In the raster shown in panel B of Figure 5, each dot corresponds to a gaze-following or joint-attention saccade produced by three viewer monkeys (D, P, and C) starting from the eye or head of M4. Note that for all the videos, gaze-following and joint-attention saccades could occur at almost any time during the video, except for 699 frames out of 6,750 (or 10.4% of the video frames) when a gaze-following or joint-attention saccade count not be induced because the face of the one of the video monkeys was not visible or the monkeys were facing the viewer (3D). The gap in gaze-following and joint-attention saccades around frame 240 represents an instance when such saccades were not possible for 10 frames.

The small cyan, majenta, and purple circles mark the frame and trial number of the example saccades shown in Figure 5E. A grand average of the temporal distribution of the saccades originating from M4 (lipsmacking) in the interaction with every other threatening partner (M1, M2, and M3) is shown in Figure 5C. The clustering of significantly increased probability of gaze-following and joint-attention saccades shows a striking similarity across the three viewers, indicating that all three viewers followed the gaze of the lipsmacking M4. This pattern may suggest a degree of automaticity, potentially driven by visual cues, but such conjecture is defeated by the low likelihood that the observers produce the same saccades in response to the same movie segment. In theory viewers could produce a maximum of 4–5 JAGF saccades per second. Even in clusters with a high probability of gaze-following and joint-attention saccades, the frequency does not exceed 1.5 JAGF saccades per second (Viewer C: 1.03 ± 0.416 Hz, *n* = 32 clusters; Viewer D: 1.14 ± 0.37 Hz, *n* = 37 clusters; and Viewer P: 1.36 ± 0.43 Hz, *n* = 17 clusters). It is unlikely, therefore, that the videos contain “irresistible” signals that trigger automatically stereotypical behaviors. For example, the raster in Figure 5 contains 1,453 JAGF saccades from 648 trials and the cluster at frame 255 contains only 128 JAGF saccades. Thus, even in this cluster, a gaze-following or joint-attention saccades was elicited in fewer than 20% of trials. It is possible that the viewer was not looking at the video when the critical frame was shown, which would reduce the opportunity of making a gaze-following or joint-attention saccade by at least 50% of the trials (note that only sessions with more than 50% viewing time of the videos were included in the analysis).

While these peaks may plead for the reflexive nature of gaze-following and joint-attention saccades, the large individual differences between the viewers argue against automaticity. Clusters of significant increases in the count of gaze-following and joint-attention saccades contributed to by all three viewers were infrequent. Frames from significant clusters of JAGF saccades overlapped only 1.3% of the time across the three observers and 7.96% of the time across two of the three observers, therefore 90.7% of the time the clusters were unique to each observer. For example, Figures 5B,C shows that the subordinate monkey produced clustering of JAGF saccades at the beginning of the video *only in observer D*. Indeed, only one of the four hot spots found in response to this video monkey was produced by more than one observer. Of all the clusters, this was the only overlapping hot spot produced by all three observers. In addition, two of the three observers watched a stimulus set with 4 male monkeys. The frames that contained their gaze-following and joint-attention saccades overlapped 3.15% of the time, and 96.8% of the time the frames were unique to each observer. In addition to differences in mentalizing, other factors may also account for these observations (early life experience, genetic differences, gender, age).

Directed gaze can be a strong trigger for gaze-following (Bettle and Rosati, 2019), so we examined periods of time when the video monkeys were facing the observer (scored as 3D) and the subsequent frames when the video monkey turned to face the direction of the conspecific in the video. In the 18 videos, there were 26 periods with 3D frames followed by a simulated interaction between the two video monkeys. 8 of these were followed by a cluster of JAGF saccades in at least one observer (31%), suggesting that the simulated directed gaze was a trigger for those JAGF saccade clusters. This finding brings together the two paradigms of interacting with a partner that could trigger gaze following: directed gaze following (as in Bettle and Rosati, 2019) vs. gaze following as a third-party observer (as in Klin et al., 2002).

Finally, we documented individual differences in the overall tendency of each viewer to make JAGF saccades. Specifically, viewer C watched 18 videos (3 sets of interacting monkeys) in which we identified 32 clusters spanning an average duration of 29.3 frames. In 18 videos watched by viewer D, 37 clusters were identified with an average duration of 36.1 frames. The viewing pattern of viewer P, although she only watched 12 videos, contained 17 clusters with the average duration of 47.7 frames. We found that a similar number of clusters were formed by saccades that originated from the dominant (*n* = 44) and subordinate (*n* = 42) monkeys, therefore the perceived social status of the observed individual may not be a factor in clustering. Overall, 89.9% of the clusters occurred during the frames that contained facial expressions.

### Bi-directional joint-attention: Viewers engage in sequences of check-back saccades between observed individuals

**Figure 6:**
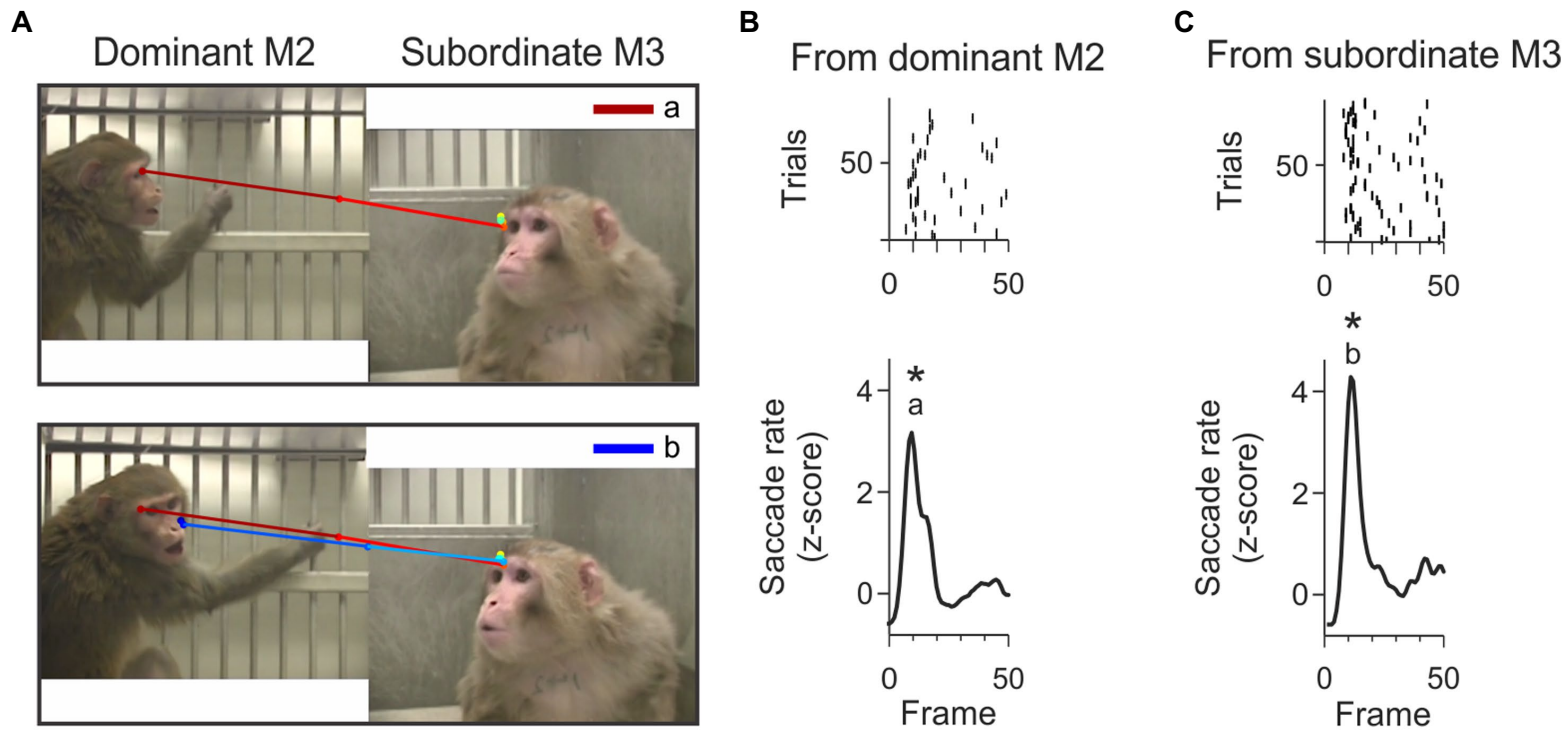
Example check-back formed by a short sequence of joint-attention saccades. **(A)** The top panel shows the first joint-attention saccade that is part of this example check-back. This saccade starts at the eyes of M2 and lands on the eyes of M3. This joint-attention saccade falls in the peak labeled with letter ‘a’ in panel B. The bottom panel shows the second joint-attention saccade starting from the eyes of M3 and landing on the sight-line of M2. This second joint-attention saccade falls in the peak labeled with letter ‘b’ in panel C. The saccades are color-coded from red (start) to dark blue (end). **(B)** Rasters (top) and smoothed histogram (bottom) of joint-attention and gaze-following saccades made by viewer P originating from M2 (dominant monkey) to M3 (subordinate monkey) in the first 50 frames of the video. Joint-attention and gaze-following saccades are clustered within 10 frames. Stars (*) indicate significant clustering at *p* < 0.05. The letter ‘a’ indicates that the first joint-attention saccade of the example check-back illustrated in panel A (top) is contained in this cluster. **(C)** Rasters (top) and smoothed histogram (bottom) of joint-attention and gaze-following saccades made by observer P from subordinate monkey M3 to dominant M2 in the first 50 frames of the video. Letter ‘b’ identifies that the saccade in panel A (bottom) is contained in this cluster. The clusters of joint-attention and gaze-following saccades from M3 to M2 and back to M3 occur within 400 ms.

A more complete understanding of an ongoing social interaction requires frequent shifts of attention from one social partner to the other. Although our experimental context did not include direct interaction with any of the observed individuals, we observed bi-directional gaze interactions that appeared similar to the “check back” looking patterns documented in chimpanzees (Call et al., 1998; Tomasello et al., 2001). Check-backs in chimps occurred in situations that required them to understand whether obstacles were in the line of sight of a demonstrator. In our third-party observers, check-backs were rapid shifts of attention between two observed individuals, that could have been used to assist the viewer’s assessment of the ongoing interaction (e.g., who is threatening whom, how does the threatened individual respond, etc.). These check-backs were identified as pairs of joint-attention saccades, back and forth between the video monkeys. All three viewer monkeys showed these types of check-backs. Of the 10,558 joint attention saccades, 2024 saccades (19.2%) formed check-back sequences. An example of a dominant-subordinate-dominant check back is shown in Figure 6A.

We determined whether the check-backs depended on the inferred dominance status of the video monkeys. In all three viewer monkeys, we found that check back sequences were more likely to originate from the dominant monkey (i.e., dominant to subordinate and back to dominant). Of all JA saccades originating from the dominant, 22.1% were the first saccade of a check back sequence. By comparison, only 16.4% of all JA saccades originating from the subordinate were the first saccade of a check back sequence (check-back/all JA saccades, monkey D: dominant 472/1923, subordinate 265/1977, chi-square test of proportions *p* < 1e-12; monkey P: dominant 336/1499, subordinate 339/1748, *p* < 0.05; monkey C: dominant 352/1601, subordinate 305/1810, *p* < 0.001). The higher proportion of check back sequences targeting the dominant for all three viewer monkeys, along with the higher proportion of single joint-attention saccades initiated from the subordinate, indicate that the different social status of the observed individuals informs these gaze behaviors.

We found that many check-backs fell within the clusters of temporally aligned JAGF saccades (hot spots). Figure 6A illustrates the scanpath of an example check-back originating from the dominant animal. In this example, the dominant monkey M2 was paired with the subordinate M3. In the first 50 frames of this video, viewer monkey P made reliable joint-attention saccades from M2 and M3 forming clusters in overlapping frames, shown as peaks in Figures 6B,C. These overlapping clusters are partially the result of JA saccades that were part of check-backs.

**Figure 7:**
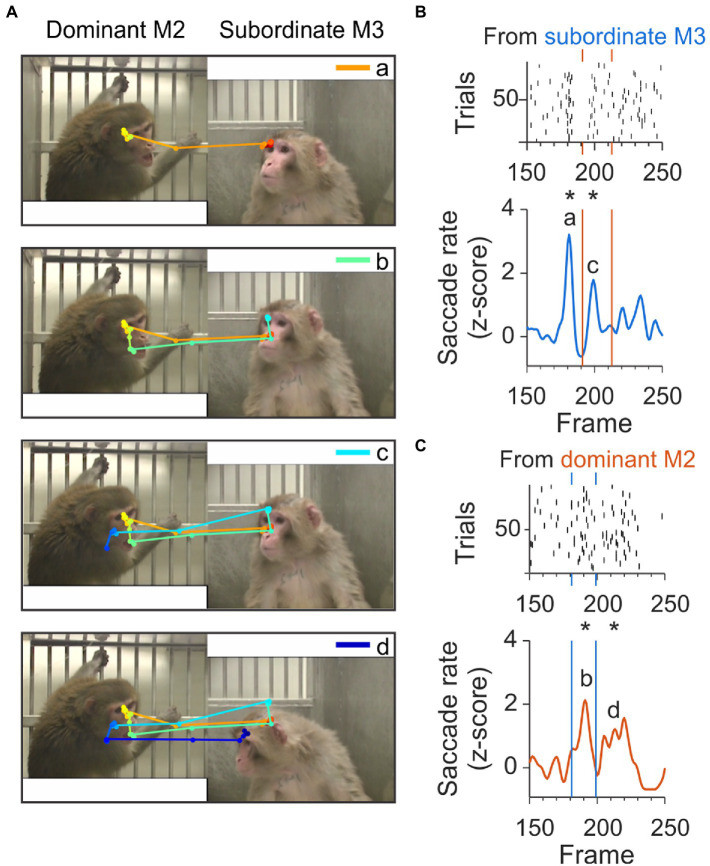
Example check-back formed by a long sequence of joint-attention saccades. **(A)** A set of subsequent joint-attention saccades that comprise multiple check-backs, starting from the eyes of M3 and ending at the eyes of M2 (saccade a, orange), continuing back to M3 (saccade b, green), then back to M2 (saccade c, light blue), and again back to M3 (saccade d, dark blue). The entire scanpath trajectory is color-coded from red (start) to blue (end). **(B)** Rasters (top) and smoothed histogram (bottom) of joint-attention and gaze-following saccades made by viewer C from subordinate monkey M3 to dominant M2 in frames 150–250 of the video. Joint-attention and gaze-following saccades are clustered at frames 180 and 200, with a gap of saccades between the clusters. Stars (*) indicate significant clustering at *p* < 0.05. Letters ‘a’ and ‘c’ identify the saccades in panel A. Orange vertical lines indicate the frames with clusters of saccades starting from the partner M2. **(C)** Rasters (top) and smoothed histogram (bottom) of joint-attention and gaze-following saccades made by observer C from dominant monkey M2 to M3. Saccades are clustered at frames 190 and 210, with a gap of saccades before and between the clusters. Stars (*) indicate significant clustering at *p* < 0.05. Letters ‘b’ and ‘d’ identify the saccades in panel A. Blue vertical lines indicate the frames with clusters of saccades starting from the partner M3.

We also found clusters that contained sequences of check-backs separated by approximately 400 ms and spanning up to 2.5 s, as shown in Figure 7. An example scanpath spanning 40 frames and multiple check back saccades is shown in Figure 7A. In this sequence, the first JA saccade originating from the subordinate monkey (shown in orange in Figure 7C, top panel) contributed to the peak ‘a’ at frame 180 in Figure 7B. This initial JA saccade was followed at frame 190 by a JA saccade (shown in green in Figure 7A second panel), originating from the dominant and contributing to peak ‘b’ in Figure 7C. At frame 200, a new JA saccade contributed to the peak marked as ‘c’ in Figure 7B, corresponding to the light blue saccade line in the third panel of Figure 7A. The last component of this sequence of check-backs is part of peak ‘d’ in Figure 7C, corresponding to the dark blue saccade line in the lower panel of Figure 7A.

As the clusters emerge from precise temporal superposition across trials, they indicate a degree of stereotypy. Although all clusters contained check back behavior, suggesting reliable, repeated patterns of checking back, overall, only 22% of check back saccades fell within these temporally aligned clusters. Thus, the check back saccades are not purely reflexive in response to a certain observed behavior but instead suggest an ongoing mentalizing process.

### Repeated viewings of the same videos do not induce sequence learning expressed through anticipatory gaze-following and joint-attention saccades

**Figure 8:**
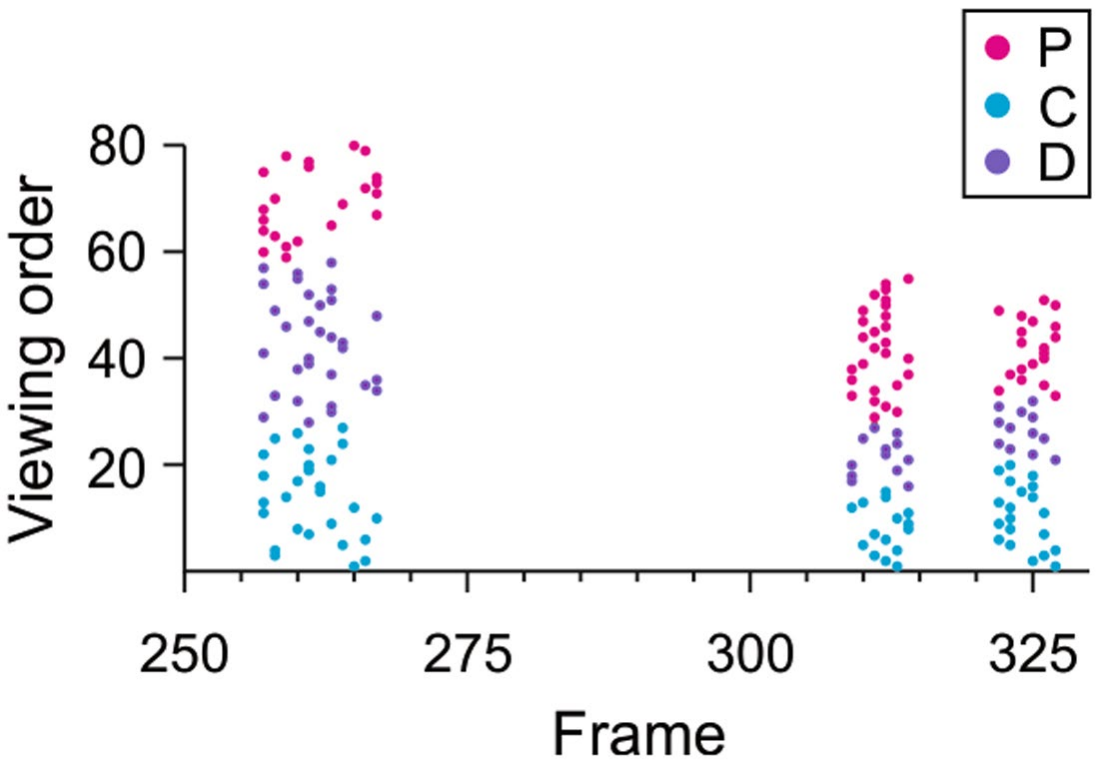
Repeated viewings do not induce anticipatory gaze-following or joint-attention saccades. The fine temporal alignment of the first saccade in each trial from 3 clusters of gaze-following and joint-attention saccades as a function of viewing order. None of the viewers showed a trend of producing earlier JAGF saccades with increased experience with the video (all Pearson correlation rho ≥0). Magenta, cyan, and purple dots indicate the JAGF saccades from viewer monkeys P, C, and D, respectively.

Some of the videos of individual monkeys were seen as many as 96 times by each viewer monkey. For example, the 15 s video showing M1 threatening was shown paired with M2, M3, and M4 that were either lipsmacking or fear grimacing. Repeated viewing of the same video can induce automatic sequence learning. In this case, the viewers might anticipate specific visual signals that reliably trigger gaze-following and joint-attention saccades. Whereas anticipatory gaze-following and joint-attention saccades could reflect predictions based on social learning, such predictions would complicate the alignment of these saccades to the behaviors shown in the videos. Using clusters of JAGF saccades, we registered the precise timing of the first gaze-following and joint-attention saccade in each trial and found no systematic relationship with the viewers’ accumulated experience with the videos. This is illustrated in Figure 8, which shows that there was no correlation between the viewing order of the videos and the timing of the first JAGF saccade in these three example clusters (Pearson correlation *p* > 0.05). Across all clusters, none of the three viewer monkeys had a systematic negative shift in the timing of their JAGF saccades over time (Pearson correlation viewer C mean rho = 0.03, *n* = 32, t-test *p* = 0.3; D mean rho = 0.08, *n* = 37, t-test *p* = 0.1; P mean rho = 0.1, *n* = 17, t-test *p* = 0.048). Thus, the first JAGF saccade of a cluster was not systematically earlier for later viewings, suggesting that the viewers responded to the content of the video rather than anticipating the events based on sequence memory.

## Discussion

Here we present evidence that gaze following and joint attention can be reliably elicited in macaques that view synthetic social interactions. We report that the scanpaths of third-party viewers of simulated conflict contain information about the content of the videos but also about the social cognitive processes of the viewer. Video frames that contained facial expressions were more effective in triggering joint attention than frames that contained neutral behaviors. Moreover, joint-attention saccades were initiated more frequently from the monkey that displayed subordinate behaviors. JAGF saccades were clustered in hot spots that corresponded to video segments that depicted exchanges of facial displays. Joint-attention saccades often appeared in sequences of back- and-forth between the monkeys (check-backs), marking the receiving-emitting cycles of social signals. Together these results suggests that gaze following and joint attention (as operationally defined for non-human primates) are not merely reflexive behaviors but are informed by higher cognitive processes akin to mentalizing.

We also show that the immediate triggers of these gaze behaviors are complex and subject to individual variation, and thus many questions related to gaze following and joint attention remain unanswered. Our approach highlighted some stimulus features that reliably trigger gaze following and joint attention but also raised new questions. It is unclear how the viewers monkeys might use the information gained through JAGF saccades and check-backs, or how these findings relate to the more substantial human literature on this topic. Although we were unable to delineate the contribution of reflexive vs. mentalizing processes to the observed gaze behaviors, this paradigm offers the possibility of interrogating the brain to directly decode the information gained and used by third-party observers. The small number of subjects used in this study was sufficient to establish this behavioral paradigm for neurophysiological recordings, but they remain insufficient to generalize our findings and make broad statements about all gaze-mediated components of social interactions among all macaques (Simons et al., 2017).

The most prominent, socially relevant, aspect of our paradigm was the inferred dominance status (or the reputation) of each stimulus monkey. The highest status animals in these pairwise interactions were always threatening, the lowest status animals were always affiliative, and the animals in the middle of the hierarchy appeared threatening or affiliative depending on the status of their social partners. We did not test whether the viewers understood the suggested hierarchy; our goal was only to add a naturalistic dimension to the otherwise artificial stimuli. The results presented here indicate that in this behavioral context, gaze following and joint attention show sensitivity to this aspect of the stimuli. The preferential origin of joint attention saccades from the subordinate animals and targeting the dominant, and the preponderance of check back saccades also targeting the dominant, argue in favor of social cognitive, rather than mere reflexive mechanisms. It is possible, indeed likely, that certain behaviors of the observed animals, i.e., stereotypical facial and bodily displays, carry “signal value” that may not only attract visual attention but also trigger JAFG saccades. This argument is strengthened by the observation that in all three observers gaze-following and joint-attention saccades were clustered in response to the same behavior, even when the observed animal was interacting with a different social partner. The reflexive nature of these gaze-following and joint-attention events is challenged, however, by the observation that they occurred in less than 10% of cases when the videos offered an opportunity for gaze following or joint attention. Remarkable individual differences, where the gaze of an individual was followed only when paired with a specific partner, further weaken the idea of automaticity. It is possible that the automatic or reflexive gaze following is favored by more schematized paradigms (e.g., Emery et al., 1997; Ferrari et al., 2000; Deaner and Platt, 2003), or paradigms where the observed individual directly engages the observer. More naturalistic stimuli, with multiple participants, might engage social cognitive processes that evolved to solve the complex challenges faced by social primates (Fujii et al., 2009; Gothard et al., 2018; Miller et al., 2022).

What caused, then, the temporal alignment of some gaze-following and joint-attention saccades? They appear to have been triggered by a specific cue contained in a small cluster of video frames. We eliminated the possibility that the cue was related to motion, as the visual flow field in the videos was uncorrelated with a higher probability of gaze-following and joint-attention saccades (see Figure 5). In direct interactions, gaze following can be cued by communicative signals such as eye contact or calling the social partner’s name (Senju and Csibra, 2008; Téglás et al., 2012). Indeed, we found that clustered JAGF saccades often followed a simulated direct gaze by one of the video monkeys, indicating a linking behavior between direct and third-party gaze following. Böckler et al. (2011) showed that eye contact inferred by a third-party observer of two individuals looking at each other can have the same cueing effect. It is possible, therefore, that our observers were cued by similar percepts. It is remarkable that some of these clusters are generated by the rapid back-and-forth saccades between the two interactive monkeys. It is as though the third-party viewer’s gaze actively deciphers the observed social exchange, bringing their social experience to the plight of the interactive parties, similarly to what has been elegantly captured by Klin et al. (2002) in humans.

Facial expressions, especially fearful faces are known to strongly modulate gaze following and joint attention (Teufel et al., 2010). Enhancements of gaze-following were obtained from macaques who followed the gaze of human demonstrators with fearful facial expressions (Goossens et al., 2008; Teufel et al., 2010). In humans, searching for a threatening target looked at by the fearful individual compels third-party observers to produce gaze-following saccades originating from the fearful face (Kuhn and Tipples, 2011). We replicated this finding in non-human primates, and we offer an important additional detail: all three viewers showed more joint-attention than gaze-following saccades starting from the subordinate monkeys. Compared to gaze following when the viewer merely looks in the same general direction, joint attention lands the viewer’s gaze on the object most likely explored by the observed individual. This attests to the motivation of the viewer to learn what the observed individual is looking at. There is a clear benefit to paying attention to what an appeasing, possibly fearful conspecific pays attention to. By following the gaze of the appeasing subordinate, the viewer gathers information about the source of the threat that motivates the appeasing behaviors. The tendency to follow the gaze of threatened subordinates in third party observers of hierarchical interactions suggests that the viewer perceives the status-driven motivational state of the observed animals, and this meets basic criteria for mentalizing (Frith and Frith, 2006; Kliemann and Adolphs, 2018).

A mentalizing component of gaze-following has been suggested by reports of human and animal studies that showed a dependence of gaze-following on the age of the demonstrator (Ciardo et al., 2014), race and power (Weisbuch et al., 2017), animal friendship (Micheletta and Waller, 2012), and the action-related expectations of the observer (Perez-Osorio et al., 2015). Furthermore, different observers may show different degrees of propensity for more reflexive or more mentalizing gaze following and joint attention, hence the large inter-individual variation. Even our three observers showed remarkable individual differences. Despite individual differences in the quantity of gaze-following and joint-attention saccades, qualitatively the three viewers showed strong similarities. All three viewers, an adult male and two adult females, made significantly more joint-attention saccades originating from face/eyes of the subordinate monkey than from the dominant monkey (Figure 4). While large individual differences may prevent convenient generalizations, they bring to light the reality of working with small, genetically, and phenotypically heterogenous populations, a feature often shared by human and non-human primate research. These differences offer opportunities to explore deeper these differences in search of tractable mechanisms (Shepherd, 2010).

Similar heterogeneous findings transpire from the literature on gaze-following abnormalities in neurodevelopment and neuropsychiatric disorders. The seminal book of Mundy and Van Hecke (2008) motivated numerous follow-up studies, many of which were focused on deficits in gaze following and joint attention in autism spectrum disorders. While some early studies claimed that a large proportion of children with autism had no deficits following the gaze of others (Leekam et al., 1998), others argued that in these children only the reflexive gaze following is preserved (Frischen et al., 2007; Nation and Penny, 2008) and that this apparently preserved faculty in autistic children relies on neural mechanisms that may be different from the mechanism that supports the same behaviors in neurotypical individuals (Greene et al., 2011). To validate this plausible conjecture, it is imperative to identify the neural underpinnings of gaze following which requires the development of naturalistic stimuli that reliably elicit gaze following during intracranial neural recordings from non-human primates. Identifying the neural circuits and the circuit dynamics that support these behaviors will serve not only a better understanding of autism but also other disorders that share with autism gaze following deficits. Among these, schizophrenia looms large, because the social deficits in these disorders are among the most treatment-resistant symptoms. Interestingly, the perception of gaze cues is more severely affected in schizophrenia than the perception of other social cues (Dalmaso et al., 2013). Likewise, the correct perception of gaze direction seems to be intact in schizophrenia, yet the social-cognitive use of such information is impaired (Palmer et al., 2018), despite a reported hyper-responsivity to gaze cues (Caruana et al., 2019). The neural underpinning of the social-cognitive processes that drive gaze-mediate social interactions, and the mechanisms that cause deficit in these behaviors are only partially understood. The stimulus set that allowed us to obtain the behaviors reported here will become instrumental for future studies that will probe the brain for a more complete understanding of these mechanisms.

Two comprehensive reviews by Emery (2000) and Shepherd (2010) and more recent empirical studies (Dal Monte et al., 2016) listed the brain areas that were implicated, based mainly on single neuron recording in non-human primates. These lists contained brain areas that showed the presence of neurons responsive faces, eyes, and gaze direction in multiple subcortical (amygdala, superior colliculus, pulvinar, hippocampus) and cortical (superior temporal sulcus, fusiform gyrus, intraparietal sulcus) areas of the brain. Both reviews emphasized that the gaze-sensitive areas are embedded in an extended social processing network concerned with face processing, attention, and action mirroring or imitation, that may all contribute to gaze following. Indeed, gaze sensitivity is necessary but not sufficient to account for the behavior itself. More detailed information was gleaned from neuroimaging studies in non-human primates trained to follow the gaze of static images (Kamphuis et al., 2009). These studies added the frontal eye fields and multiple face patches along the superior temporal sulcus (Tsao et al., 2006) to the already known lists of brain structures that might be implicated in gaze following. These areas were slightly more anterior than the gaze-following region of the posterior superior temporal sulcus identified in humans (Materna et al., 2008; Marquardt et al., 2017) emphasizing the homologies between humans and non-human primates. More recently, Kraemer et al. (2020) have shown that the human lateral intraparietal sulcus and the inferior frontal junction contribute spatial information to gaze following. While neuroimaging studies in humans can highlight the location of large groups of neurons that become active during gaze following, single neuron recording in humans and non-human primates can identify the specific contribution of the neurons in these areas to gaze following and joint attention.

## Data availability statement

The raw data supporting the conclusions of this article will be made available by the authors, without undue reservation.

## Ethics statement

The animal study was reviewed and approved by Institutional Animal Care and Use Committee.

## Author contributions

TC collected the data, coordinated the scoring of the videos, analyzed the data, and made figures. SL set up the equipment, collected the data, scored the videos, analyzed the data, and made figures. AM coordinated the analysis, carried out part of the statistical analysis, and made figures. SK and CB scored the scanpath, analyzed the data, and made figures. KG designed the experiments, supervised the analyses, and wrote the manuscript. All authors contributed to the article and approved the submitted version.

## Funding

This work was supported by the NIH/NIMH under grants R01MH121009 and P50MH100023.

## Conflict of interest

The authors declare that the research was conducted in the absence of any commercial or financial relationships that could be construed as a potential conflict of interest.

## Publisher’s note

All claims expressed in this article are solely those of the authors and do not necessarily represent those of their affiliated organizations, or those of the publisher, the editors and the reviewers. Any product that may be evaluated in this article, or claim that may be made by its manufacturer, is not guaranteed or endorsed by the publisher.

## Footnotes

^1^ https://monkeylogic.nimh.nih.gov/
